# Ligand-dependent EphA7 signaling inhibits prostate tumor growth and progression

**DOI:** 10.1038/cddis.2017.507

**Published:** 2017-10-12

**Authors:** Shibao Li, Zhiyuan Wu, Ping Ma, Yinhai Xu, Yuming Chen, Hua Wang, Ping He, Zhihua Kang, Lingyu Yin, Yao Zhao, Xinju Zhang, Xiao Xu, Xiaochao Ma, Ming Guan

**Affiliations:** 1 Department of Laboratory Medicine, The Affiliated Hospital of Xuzhou Medical University, Xuzhou, China; 2 Department of Laboratory Medicine, Huashan Hospital, Shanghai Medical School, Fudan University, Shanghai, China; 3 Department of Laboratory Medicine, Renji Hospital, School of Medicine, Shanghai Jiaotong University, Shanghai, China; 4 Department of Central Laboratory, Huashan Hospital, Shanghai Medical School, Fudan University, Shanghai, China; 5 Department of Pharmaceutical Sciences, Center for Pharmacogenetics, School of Pharmacy, University of Pittsburgh, Pittsburgh, PA, USA

## Abstract

The downregulation of receptor tyrosine kinase EphA7 is frequent in epithelial cancers and linked to tumor progression. However, the detailed mechanism of EphA7-mediated prostate tumor progression remains elusive. To test the role of EphA7 receptor in prostate cancer (PCa) progression directly, we generated EphA7 receptor variants that were either lacking the cytoplasmic domain or carrying a point mutation that inhibits its phosphorylation by site-directed mutagenesis. Overexpression of wild-type (WT) EphA7 in PCa cells resulted in decreased tumor volume and increased tumor apoptosis in primary tumors. In addition, ectopic expression of WT EphA7 both can delay PCa cell proliferation and could inhibit PCa cell migration and invasion. This protein can also induce PCa cell apoptosis that correlated with increasing the protein expression levels of Bax, elevating the caspase-3 activities, reducing the protein expression levels of Bcl-2 and facilitating the dephosphorylation of Akt, which is further increased by the stimulation of ephrinA5-Fc. However, expression of these EphA7 mutants in PCa cells has no effect *in vivo* and *in vitro*. The expression of EphA7 and ephrinA5 was significantly decreased in PCa specimens compared with BPH tissues or paired normal tissues. Moreover, the phosphorylation of EphA7 was positively related with ephrinA5 expression in human prostate tissues. In sum, receptor phosphorylation of EphA7, at least in part, suppress PCa tumor malignancy through targeting PI3K/Akt signaling pathways.

Prostate cancer (PCa) is one of the most common male malignancy, and the second leading cause of death from cancer for men.^1^ With the aging of the population and changes in diet worldwide, an increasing number of PCa cases have been reported in recent years.^1, 2^ Metastasis remains the primary cause of PCa-related death.^2, 3^ Given this fact, the traditional therapeutic modalities such as surgery, chemotherapy therapy and androgen ablation therapy are only modestly effective.^4, 5^ Therefore, understanding the molecular mechanisms contributing to PCa progression could provide insights into effective therapeutic strategies.

Eph receptors, the largest family of receptor tyrosine kinases, and their ephrin ligands not only regulate many physiological processes in the development of organs, but also have been implicated in numerous pathologies, including tumor progression.^6, 7^ Among these Eph receptors, EphA7 receptor has attracted growing attention in tumor research. Our previous studies have shown the high prevalence of EphA7 downregulation in PCa patients, which can be introduced by promoter hypermethylation.^8^ Similar results also have been observed in other human malignancies, including colorectal cancers,^9^ human germinal center B-cell lymphomas,^10^ T-cell lymphoblastic lymphomas^11^ and oral squamous cell carcinoma.^12^ Recently, deep sequencing analyses revealed many somatic driver mutations of EphA7 in small cell lung cancer, melanoma, and head and neck carcinoma.^13^ Those cancer somatic mutations were reported to span throughout the functional domains of the EphA7 receptor, and some have been shown to disrupt ephrin ligand-dependent receptor signaling.^13^ Moreover, in xenograft models of lymphomas, administration of the purified EPHA7^TR^ protein was shown to significantly inhibit tumor growth via blocking EphA2 phosphorylation and oncogenic signals.^14^ Taken together, these findings show that EphA7 receptors are tumor suppressors in the relevant tumors. However, both the biological roles and the underlying molecular mechanisms of EphA7 still need to be studied in PCa.

Here, we investigated the tyrosine site and the function of EphA7 phosphorylation using overexpression of EphA7 mutants either truncating the cytoplasmic domain or carrying a point mutation in PCa cells. We showed that ligand-dependent EphA7 signaling resulted in the downregulation of tumor volume and the enhancement of tumor cell apoptosis in primary tumors, and significantly inhibited the proliferation, migration and invasion of PCa cells, as well as induced PCa cell apoptosis, whereas ligand-independent EphA7 signaling did not have this effect. These data imply that the phosphorylation of EphA7 receptor, at least in part, suppresses PCa malignancy through targeting PI3K/Akt signaling pathways.

## Results

### Cell density regulates EphA7 receptor phosphorylation in LNCaP cells

**Figure 1:**
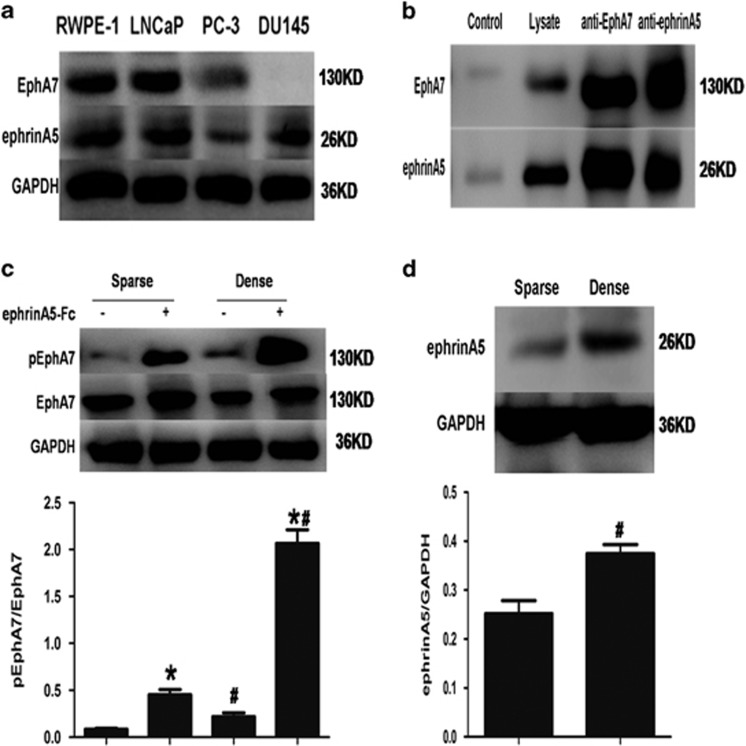
The phosphorylation of EphA7 receptor was regulated by cell density. (**a**) The protein expression of EphA7 and ephrinA5 was analyzed by western blotting in four prostate cell lines. (**b**) The interaction between EphA7 and ephrinA5 in LNCaP cells by co-immunoprecipitation and western blot analysis. (**c**) The phosphorylation analysis of EphA7 receptors by immunoprecipitation. Dense (approximately 100% confluence) and sparse (approximately 30% confluence) LNCaP cells were serum-deprived for 24 h and then stimulated with 1.0 *μ*g/ml ephrinA5-Fc or Fc for 15 min. The EphA7 protein was enriched using immunoprecipitationwith anti-EphA7 antibody and then divided in two aliquots for immunoblotting with anti-EphA7 antibody and anti-phosphotyrosine (4G10) antibody. (**d**) Western blot analysis of expression of the ephrinA5 ligand. Data are shown as the mean±S.D. *P<0.05, *versus* the corresponding LNCaP cells without the stimulation of ephrinA5-Fc; ^#^*P*< 0.05, *versus* the correspondingly sparse LNCaP cells

Eph receptor function is dependent on the stimulation of its homologous ligand, ephrin. Prior studies have shown that the optimum ligand of EphA7 is ephrinA5.^15^ We explored whether the optimum ligand of EphA7 is also ephrinA5 in PCa, whether the phosphorylation of EphA7 receptors is related to the stimulation of endogenous ephrinA5 ligand in a cell–cell contact-dependent manner and whether the exogenous ephrinA5 ligand can further elevate the level of EphA7 tyrosine phosphorylation. The ephrinA5-EphA7 complexes were observed by co-immunoprecipitation in LNCaP cell lysates (Figure 1b), which highly expresses both the endogenous ephrinA5 ligand and the endogenous EphA7 receptor (Figure 1a). Also, the level of phosphorylated EphA7 receptor was upregulated by cell density, and was able to be further enhanced by the stimulation of exogenous ephrinA5 ligand (Figure 1c). Furthermore, with increasing cell density, the ephrinA5 expression level gradually increased, whereas no obvious difference on EphA7 expression was observed in different cell density groups (Figures 1c and d). These results showed that the tyrosine phosphorylation of the EphA7 receptor was regulated by its ligand ephrinA5 and cell density.

### Tyrosine 791 of EphA7 is the major phosphorylation site for the EphA7 receptor in PCa

**Figure 2:**
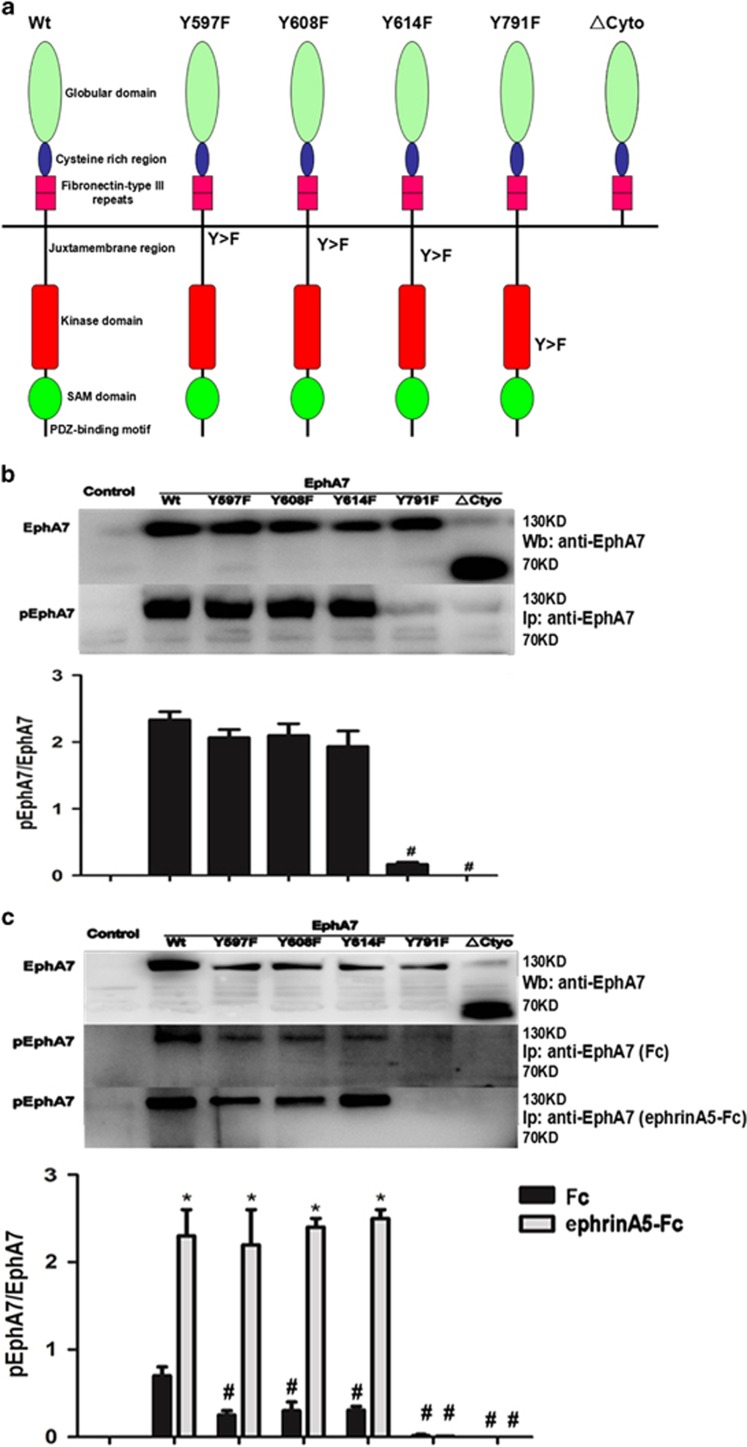
Tyrosine 791 of EphA7 is the major phosphorylation site for the EphA7 receptor in PCa cells. (**a**) A diagram of EphA7 receptor structure and mutants. (**b**) The expression and phosphorylation of EphA7 in DU145 cells stably overexpressing either WT EphA7 receptor or EphA7 mutants. (**c**) The expression and phosphorylation of EphA7 in PC-3 cells stably overexpressing either EphA7(WT) receptor or EphA7 mutants. Cells were serum-deprived for 24 h and then stimulated with 1.0 *μ*g/ml of ephrinA5 ligand or Fc for 15 min. After cell lysis, EphA7 was immunoprecipitated with anti-EphA7 antibody and then divided into two aliquots for immunoblotting with anti-EphA7 antibody and anti-phosphotyrosine (4G10) antibody. Data are shown as the mean±S.D.^*#*^*P*<0.05, *versus* the respective WT EphA7 group, *P<0.05, *versus* the respective control group

To explore the role of EphA7 receptor phosphorylation, we first predicted several potential phosphorylated sites of tyrosine via sequence analysis (www.phosphositeplus.org) and generated the human wild-type (WT) EphA7 cDNA and the following EphA7 mutants: Y597F/Y608F/Y614F in the juxtamembrane domain (DM); Y791F in the kinase domain (KD); and the ΔCyto mutant lacking the entire cytoplasmic region (Figure 2a). The resultant mutants were tested in PCa cell lines (PC-3, and DU145), which either express very low levels of endogenous EphA7 or do not express endogenous EphA7. As shown in Figures 2b and c, the phosphorylated EphA7 receptor was detected in the WT EphA7 and DM-mutant group, whereas overexpression of the KD mutant or ΔCyto forms of EphA7 failed to confer the phosphorylation of EphA7 receptor in EphA7-deficient cells, implying that the tyrosine 791 of the cytoplasmic domain is critical to EphA7 receptor phosphorylation. Interestingly, no endogenous EphA7 protein was observed using immunoprecipitation in PC-3 cells, which expressed extremely low levels of EphA7 protein in Figure 1a. This discrepancy may be the contribution of low sensitivity of immunoprecipitation.

Then, we analyzed the EphA7 receptor phosphorylation in response to ephrinA5-Fc treatment in PC-3 cell lines. Data showed that the overexpression of DM or WT of the EphA7 mutant resulted in a substantial enhancement of ligand-independent EphA7 receptor phosphorylation, which was further enhanced by the ephrinA5-Fc treatment, implying that the phosphorylation of EphA7 receptor required the stimulation of ephrinA5-Fc (Figure 2c). Taken together, these data suggest that the primary phosphorylation site of EphA7 is tyrosine 791, and dependent on the stimulation of ephrinA5 ligand.

### Ligand-dependent inhibition of tumor cell growth by EphA7 in PCa requires Y791 phosphorylation

**Figure 3:**
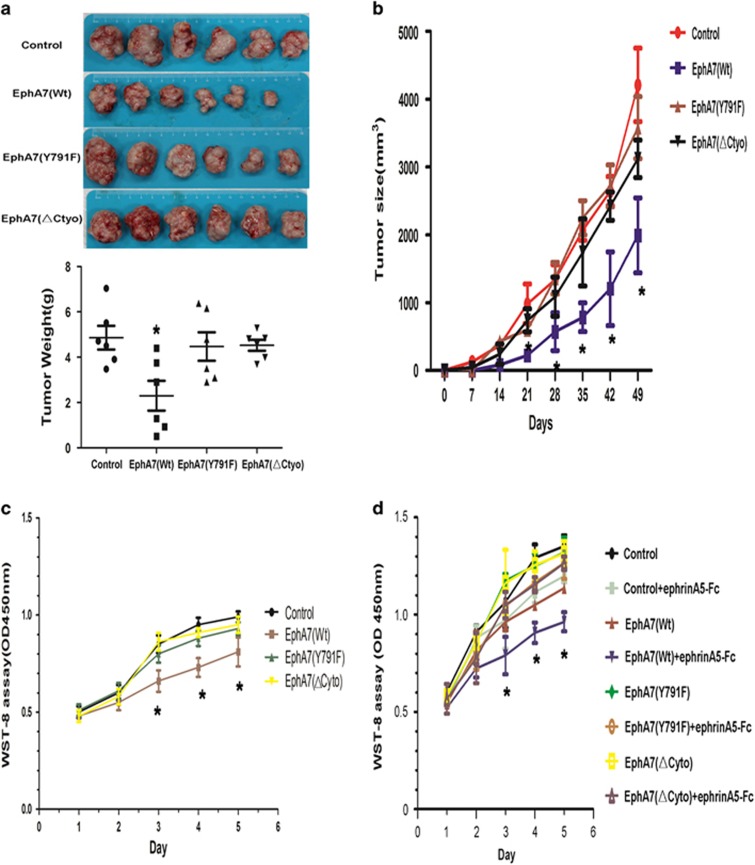
WT EphA7 inhibit tumor growth *in vitro* and *in vivo*. (**a**) Subcutaneous xenograft PCa models were generated using PC-3/Control, PC-3/EphA7(WT), PC-3/EphA7(KD), or PC-3/EphA7(ΔCyto) cells. After mice had been terminally narcotized and killed over 49 days, tumors were removed and the weights measured. The average horizontal lines represent the means in the groups. (**b**) Tumor growth was monitored over seven weeks, and the average tumor volumes of primary tumors were calculated (six mice per group). (**c**) The proliferation of DU145 cells stably overexpressing either EphA7(WT) receptor or EphA7 mutants was evaluated using a colorimetric WST-8 assay. (**d**) Proliferation of PC-3 cells overexpressing the EphA7(WT) receptor or EphA7 mutants. Cells were plated in 96-well plates and then serum-deprived for 24 h. After stimulation of 1.0 *μ*g/ml of ephrinA5 ligand or Fc, cell viability from 24 h (To) and seven days (T7) in culture was determined using a colorimetric WST-8 assay. Data are shown as the mean±.S.D. *P<0.05, *versus* the respective control group

To evaluate whether the phosphorylation of the EphA7 receptor could affect tumor growth, we first analyzed the effect of different EphA7 mutants forms on tumor growth *in vivo*. A subcutaneous xenograft model was established using PC-3/Control, PC-3/EphA7(WT), PC-3/EphA7(KD) or PC-3/EphA7(ΔCyto) cells in nude mice. The primary tumors of PC-3/EphA7(WT) grew slower than the PC-3/Control tumors, PC-3/EphA7(KD) tumors and PC-3/EphA7(ΔCyto) tumors. However, there was no significant difference in tumor growth rate between the PC-3/Control tumors and the PC-3/EphA7(KD) tumors, as well as the PC-3/EphA7(ΔCyto) tumors (Figure 3b). Similarly, the average tumor weights of the PC-3/EphA7(WT) primary tumors were significantly less than those of the PC-3/Control tumors, the PC-3/EphA7(KD) tumors and the PC-3/EphA7(ΔCyto) tumors (*P*<0.05) (Figure 3a). The above results suggest that WT EphA7 receptor inhibit prostate tumor growth *in vivo*.

Next, PCa cells proliferating ability was detected in DU145 cells and PC-3 cell lines. As shown in Figures 3c and d, compared with control cells, the capacity of cell proliferation was slowing-down in overexpression of WT EphA7 cell, which was further aggravated by the ephrinA5-Fc administration in PC-3 cells that express very low levels of endogenous ephrinA5. In contrast, stably transfected PCa cells with the KD mutant or ΔCyto forms of EphA7 did not show any significant difference in cell proliferation. Similar results have been observed by Ki-67 IHC staining of primary tumors in a subcutaneous xenograft model (Supplementary Figure S1). These intriguing results suggest that the suppressive effect of EphA7 receptor is dependent on the phosphorylation of tyrosine 791 and on the stimulation of the exogenous ephrinA5 ligand.

### Ligand-dependent inhibition of cell migration and invasion by EphA7 in PCa requires Y791 phosphorylation

**Figure 4:**
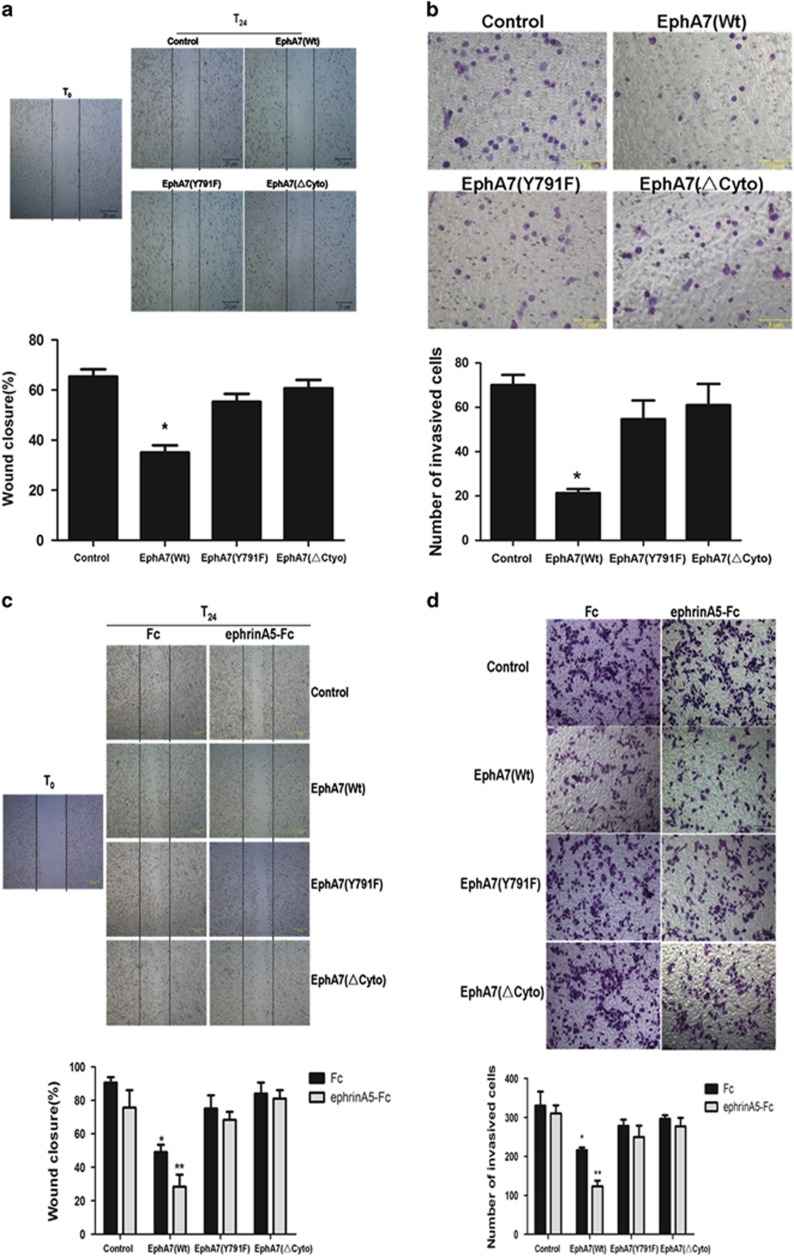
Ligand-dependent inhibition of cell migration and invasion by EphA7 in PCa. (**a**) DU145 cell migration activity was measured using the wound healing assay. (**b**) DU145 cell invasion activity was detected with the Matrigel invasion assay (× 100). (**c**) PC-3 cell migration activity was measured using the wound healing assay. Cells were serum-deprived for 24 h, scratched with a tip and photographed (T0). After stimulation of 1.0 *μ*g/ml of ephrinA5 ligand or Fc for 15 min, cells were cultured in complete medium for 24 h and photographed (T24). (**d**) PC-3 cell invasion activity was detected with the Matrigel invasion assay (× 100). A total of 5 × 10^4^ cells were plated in transwell inserts, treated with 1.0 *μ*g/ml of ephrinA5 ligand or Fc for 15 min and cultured for 24 h. All experiments were performed in triplicate. Data are shown as the mean±S.D. *P< 0.05 *versus* the respective control group, **P<0.05 *versus* the respective EphA7(WT) expression group

Then, we assess the effects of EphA7 phosphorylation on the ability of PCa cells to migrate and invade using the scratch migration and Matrigel invasion assays. Migratory ability was significantly decreased in DU145/EphA7(WT) cells compared with DU145/EphA7(Control) cells, DU145/EphA7(KD) cells and DU145/EphA7(ΔCyto) cells (Figure 4a). PC-3/EphA7(WT) cells showed a slight reduction in migration, but the stimulation of ephrinA5-Fc significantly decreased cell migration, whereas PC-3/EphA7(KD) cells and PC-3/EphA7(ΔCyto) cells were unaffected (Figure 4c). Similarly, the Matrigel invasion assays displayed that the number of invasive cells was significantly reduced in the EphA7(WT) group compared with control and EphA7(KD) groups, as well as EphA7(ΔCyto) group, and was further decreased by the ephrinA5-Fc administration in the PC-3/EphA7(WT) group (Figures 4b and d). These intriguing results suggest that the suppressive effect of EphA7 receptor on tumor migration and invasion is dependent on the phosphorylation of tyrosine 791 and the stimulation of ephrinA5 ligand.

### Tyrosine 791 phosphorylation of EphA7 induces prostate carcinoma cell apoptosis

**Figure 5:**
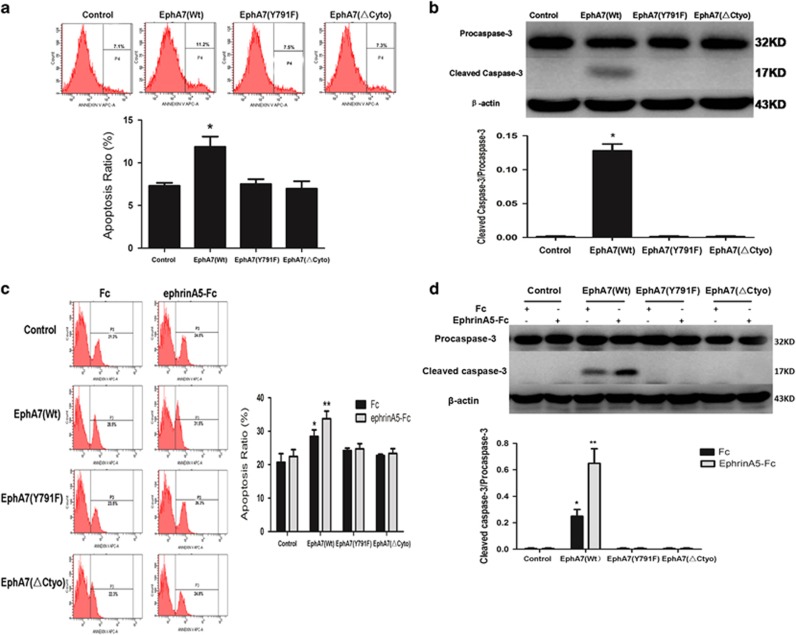
Ligand-dependent promotion of cell apoptosis by EphA7 in PCa. (**a**) DU145 cell apoptosis was analyzed by FACS after Annexin V-APC staining. (**b**) Western blot analysis of procaspase-3 and cleaved caspase-3 expression in DU145 cell-overexpressing EphA7(WT) receptor or EphA7 mutants. (**c**) PC-3 cell apoptosis was analyzed by FACS after Annexin V-APC staining. A total of 5 × 10^6^ PC-3 cells overexpressing EphA7(WT) receptor or EphA7 mutants were serum-deprived for 24 h, and treated with 1.0 *μ*g/ml of ephrinA5 ligand or Fc for 15 min. Then, cells were stained using Annexin V-APC and quantified by FACS. (**d**) Western blot analysis of procaspase-3 and cleaved caspase-3 expression in PC-3 cells overexpressing EphA7(WT) receptor or EphA7 mutants. PC-3 cells overexpressing EphA7(WT) receptor or EphA7 mutants were serum-deprived for 24 h, treated with 1.0 *μ*g/ml of ephrinA5 ligand or Fc for 15 min and lysed. Then, cell lysate was immunoprecipitated with anti-procaspase-3 antibody and anti-cleaved caspase-3 antibody. Data are shown as the mean±S.D. *P<0.05 *versus* the control group. **P<0.05 *versus* the respective EphA7(WT) expression group

Next, we investigated whether tyrosine 791 phosphorylation of EphA7 can induce PCa cell apoptosis. Compared with the control and mutant groups, the PCa cell apoptosis rate was significantly increased in the WT EphA7 group (*P*<0.01, Figures 5a and b). Western blot determination also indicated that WT EphA7 upregulated cleaved caspase-3 protein, which was further increased by ephrinA5-Fc administration (Figures 5c and d). Meanwhile, as expected, the number of apoptosis cells significantly increased in WT EphA7 expressing tumors compared with the control group by active-caspase-3 staining *in vivo*, whereas the EphA7 mutant-expressing tumors, either EphA7(KD) or EphA7(ΔCyto), showed little or no apoptosis (Supplementary Figure S1). These data support the idea that EphA7-inducing apoptosis requires the kinase-dependent signaling of EphA7 receptor.

### Involvement of the PI3K/Akt signaling pathway in tyrosine 791 phosphorylation of EphA7-induced cell apoptosis in PC-3 cells

**Figure 6:**
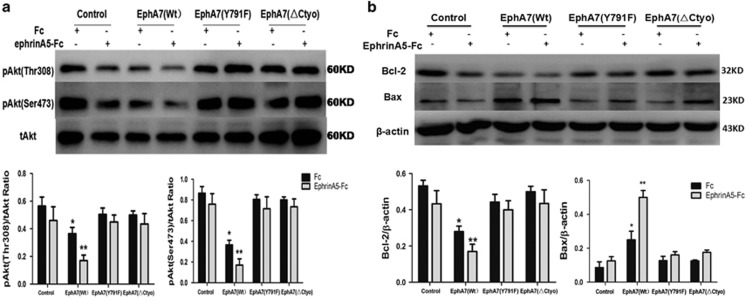
Ligand-dependent EphA7 signal influences the expression levels of pAkt(Thr308), pAkt(Ser473), Bcl-2 and Bax in PC-3 cells. PC-3 cells, overexpressing EphA7(WT) receptor or EphA7 mutants, were cultured under the fetal bovine serum-free RPMI-1640 media for 24 h, treated with 1.0 *μ*g/ml of ephrinA5 ligand or Fc for 15 min, and lysed. Cell lysate was immunoprecipitated with anti-pAkt(Thr308) antibody, anti-pAkt(Ser473) antibody and anti-tAkt antibody (**a**), as well as anti-Bcl-2 antibody, anti-Bax antibody and anti-*β*-actin antibody (**b**). All experiments were performed in triplicate. Data are shown as the mean±S.D. *P< 0.05 *versus* the control group. **P< 0.05 *versus* the respective EphA7(WT) expression group

The PI3K/Akt signal pathway participated in the regulation of cell proliferation, survival and apoptosis via various downstream effectors, such as the Bcl-2 family. Alterations in the expression levels of PI3K/Akt signaling molecules were evaluated in PC-3 cells post-transfection with EphA7 for 48 h. Compared with the control group, the level of pAkt and Bcl-2, an anti-apoptotic protein, was significantly reduced in PC-3/EphA7(WT) cells, whereas the elevated protein level of Bax was observed in PC-3/EphA7(WT) cells, which was able to be further increased by treatment of ephrinA5-Fc. In addition, the expression levels of total Akt were unchanged between the control cells and PC-3/EphA7(KD) cells, as well as PC-3/EphA7(ΔCyto) cells (Figure 6). These results suggest that EphA7-induced PCa cell apoptosis may be closely related to the PI3K/Akt signaling pathway.

### The phosphorylation of EphA7 is positively related with ephrinA5 expression in human prostate tissues

To investigate whether the phosphorylation of EphA7 is correlated to ephrinA5 expression in clinical samples, we first used real-time PCR analysis to examine the mRNA level of EphA7 and its ligand ephrinA5 in 50 benign prostate hyperplasia (BPH) tissues and 64 PCa specimens. Compared with BPH tissues or paired normal tissues, 45.3% (29/64) PCa samples and 60.9% (39/64) PCa tissues, respectively, displayed the downregulation of EphA7 mRNA and the decline of ephrinA5 transcript. The association analysis between clinicopathologic parameters and EphA7 mRNA expression, as well as ephrinA5 transcript level showed that the level of ephrinA5 transcript was negatively correlated with Gleason score (*P*=0.013) and TNM staging (*P*=0.016; Table 1).

**Figure 7:**
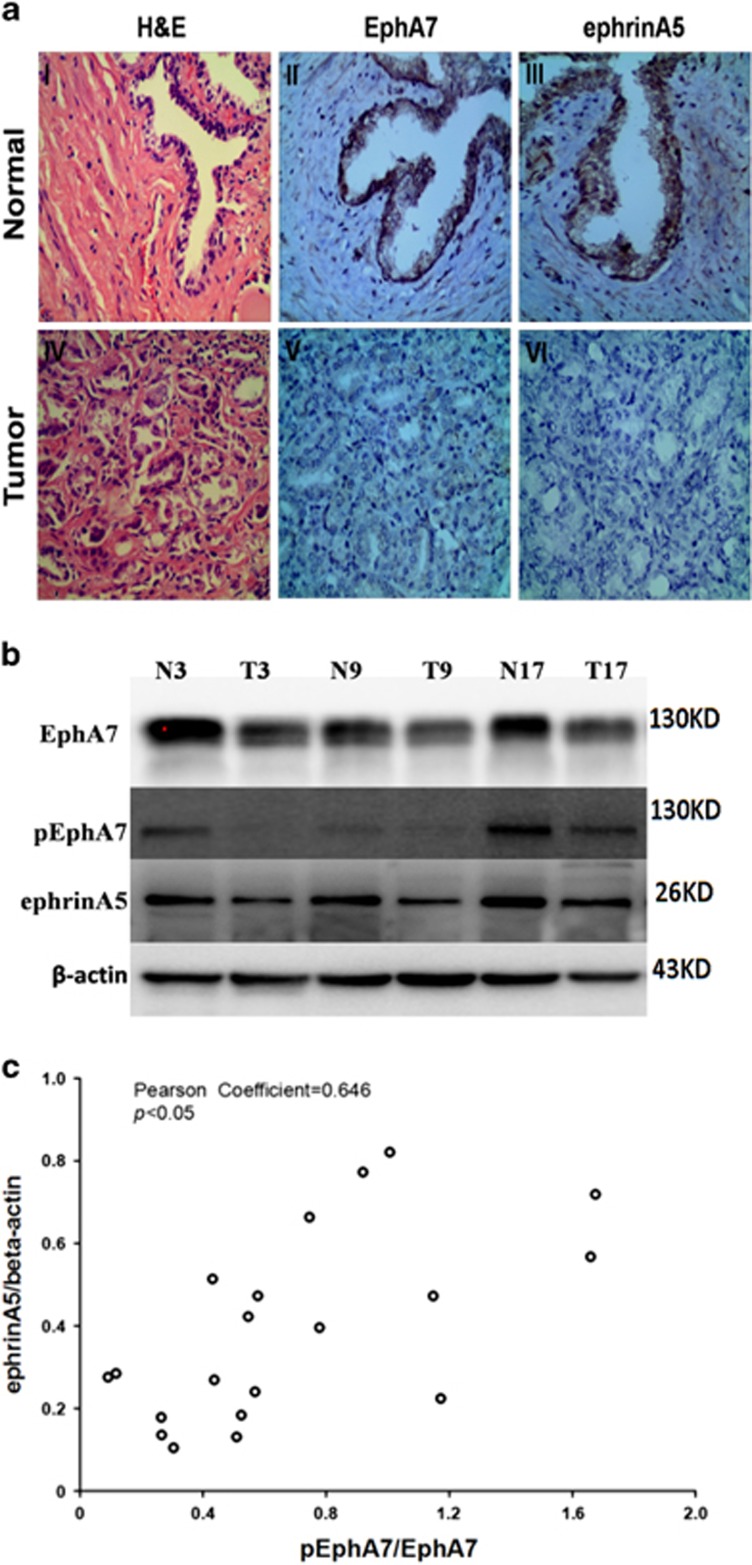
The phosphorylation of EphA7 receptor and the expression of EphA7 and ephrinA5 in human prostate tissues. (**a**) Representative photographs of EphA7 and ephrinA5 immunostaining in prostate tissue. (I–III) The adjacent non-cancerous prostate tissue. (IV–VI) The prostate carcinoma tissue (magnification, × 400). (**b**) Representative phosphorylation analysis of EphA7 receptors and the expression of EphA7 and ephrinA5 in human prostate tissues. (**c**) pEphA7/EphA7 correlates with ephrinA5 expression. Data are shown as the mean±S.D.

We next analyzed the protein expression of ephrinA5 and EphA7, as well as the phosphorylation of EphA7 in 20 pairs PCa and normal tissues by immunohistochemistry (IHC) staining or western blotting. Representative IHC results displayed that immunostaining of the EphA7 and ephrinA5 protein could be observed in the cytoplasm of all paired non-cancerous tissues. Among the 20 prostate carcinoma specimens, 13 (65.0%) exhibited undetectable or weak ephrinA5 immunostaining, and 40.0% (8/20) of the tumor tissues had no intense immunostaining of the EphA7 protein (Figure 7a). Western blotting analysis displayed that the level of both EphA7 phosphorylation and ephrinA5 expression was significantly decreased in PCa tissues compared with that in normal tissues (Figure 7b), and that the expression of ephrinA5 positively correlated with the ratio of pEphA7/EphA7 in PCa tissues (Figure 7c), implying that the phosphorylation of EphA7 may be regulated by ephrinA5 in PCa progression.

## Discussion

Our previous studies showed that EphA7 is epigenetically reduced or lost in PCa and that the ectopic expression of EphA7 is involved in prostate carcinogenesis.^8^ However, the underlying molecular mechanisms involved are still ill defined. In the present study, we provide substantial evidence that EphA7, as a tumor suppressor, restrains PCa cell migration and invasion via delaying cell growth and inducing cell apoptosis in response to its cognate ligand ephrinA5. Whereas, the EphA7 deletion mutant, carrying a Y791F point mutation (KD) or lacking its cytoplasmic region (ΔCyto), does not have this effect *in vivo* and *in vitro*. This observation suggests that EphA7 receptor phosphorylation may be required for EphA7 signaling-mediated tumor progression. In our model, after ephrinA5 specifically bound to EphA7, the level of EphA7 receptor phosphorylation significantly increased and the EphA7 phosphorylation was negatively correlated with prostate carcinogenesis. Furthermore, EphA7 signaling not only greatly enhanced the apoptosis-inducing signaling molecules activity of Bax and active-caspase-3 but also inhibited the expression of anticancer molecules Bcl-2 and the phosphorylation levels of Akt, a pivotal regulated kinase of the PI3K/Akt signaling pathway. These results support the action of EphA7 as a negative regulator of PCa progression via a ligand-dependent mechanism.

As a member of the Eph receptors group, EphA7 is highly conserved in vertebrates from fish to humans,^16^ and has a sometimes paradoxical role in cancer.^17, 18, 19^ Previous studies have demonstrated that EphA7 is epigenetically silenced in the progression of many tumors, implying that EphA7 may act as a tumor suppressor.^9, 12^ Meanwhile, various other studies have confirmed that EphA7 not only is overexpressed in the stomach,^18^ pancreas^20^ and lung,^21^ but also promotes tumor development by inhibiting carcinoma cell apoptosis, increasing cancer cell proliferation, and facilitating invasion and migration, suggesting that EphA7 receptors can contribute to tumor progression.^22, 23^ The contradictory findings above prompted us to ask whether the EphA7 receptor is pro-oncogenic or anti-oncogenic in PCa. In our study, we examined the biological role of EphA7 in PCa cell lines and found that EphA7 significantly inhibited cell migratory and invasive capabilities *in vitro*. A previous microarray analysis of the gene expression profiling of LNCaP cells (androgen dependent) and LNCaP derived C4-2 cells (androgen independent) suggested that EphA7 is one of the upregulated genes in LNCaP but is not expressed in C4-2.^24^ Lee *et al.*^25, 26^ carried out a series of studies on EphA7 receptor function during early brain development and have shown that the EphA7 receptor interacts with death receptors such as tumor necrosis factor receptor 1 (TNFR1) to induce neural epithelial cell apoptosis and to decrease cell viability. In addition, they demonstrated that EphA7 negatively regulates neural progenitor cell proliferation in brain development.^25, 26^ Consistent with the previous studies,^12, 25, 26^ our study showed that the overexpression of EphA7 also significantly inhibited cell proliferation and induced PCa cell apoptosis *in vitro* and *in vivo*, which delayed the primary tumor growth *in vivo*. However, the ectopic expression of the EphA7 deletion mutant, carrying a Y791F point mutation (KD) or lacking its cytoplasmic region (ΔCyto), did not have this effect *in vivo* and *in vitro*, suggesting that the anti-oncogenic functions of EphA7 may be involved in the tyrosine phosphorylation of EphA7 kinase domain.

Classic oncogenic transformation by the Eph receptor includes elevated levels of receptor phosphorylation. However, it is not clear whether receptor phosphorylation is required for EphA7 receptor-mediated oncogenic transformation. Ligand binding triggers Eph receptor phosphorylation. Given the diverse nature of Eph–ephrin interactions, EphA7 can bind all five ephrinA ligands. However, the ephrin expression profile has its specificity in different tissues, which results in the selectivity and specificity of ephrinA ligand combined with EphA7. Noberini *et al.*^15^ carried out a study on measuring the binding of ephrin-Fc fusion proteins to Eph receptors and found that the natural ligand of EphA7 was ephrinA5 by ELISA and pull-down assays. Consistent with these data, the current study identified the interaction between EphA7 and ephrinA5 in PCa cells and revealed EphA7 tyrosine phosphorylation of PCa cells, which can be further elevated by the stimulation of ephrinA5-Fc, implying that the biological function of EphA7 is regulated by its homologous ligand ephrinA5 in PCa. Additional analysis of the downstream signaling pathways of EphA7 receptors dependent on ephrinA5 binding in PCa will be required to address this possibility.

Ligand-dependent EphA7 signaling can both inhibit cell proliferation and survival signaling and induce apoptotic cell signaling.^26, 27^ Akt, also known as PKB or Rac, is a pivotal protein kinase of the PI3K signaling pathway that regulates cell proliferation, survival and apoptosis via the downstream effectors such as the Bcl-2 family.^28^ Downregulation of Bcl-2 or upregulation of Bax may induce cell apoptosis and subsequently activate caspase-3.^22, 27^ Therefore, the present study examined the expression levels of Bcl-2 and Bax, and the activity of Akt and caspase-3, an apoptosis-associated protein. Overexpression of EphA7(WT) significantly decreased the expression of pAkt and Bcl-2, and increased the expression of Bax and the activation of caspase-3, which can be further elevated by the stimulation of ephrinA5-Fc in PC-3 cells. These results indicated that EphA7 induced PCa cell apoptosis predominantly via the PI3K/Akt signaling pathway.

This study has some potential limitations regarding the mRNA expression of EphA7 and ephrinA5 in PCa tissues. First, laser microdissection could not be performed to isolate PCa cells, thus both epithelial cells and stromal cells could have contributed to the expression of EphA7 and ephrinA5. However, our IHC analysis suggests that the majority of EphA7 protein and ephrinA5 protein expression was from epithelial cells. Second, we cannot validate the role of endogenous ephrinA5 in EphA7 receptor phosphorylation via transfection of ephrinA5 expressing vector in PC-3 cells, but the similar study has been performed in both PC-3 cells treating with soluble ephrinA5-Fc and Du145 cells expressing very high levels of endogenous ephrinA5. Furthermore, the ligand-dependent antitumor effect of EphA7 receptor was confirmed in PC-3 cell line, because only PC-3 cell lines express very low levels of endogenous EphA7 and ephrinA5 in four prostate cell lines(RWPE-1, PC-3, LNCaP and DU145). Therefore, as a more general model for PCa, more PCa cell lines should be analyzed in the future.

In conclusion, the phosphorylated EphA7 can exert potential tumor-suppressive effects in regulating the development and progression of PCa. In addition, the anticancer effect of EphA7 is dependent on the presence of the primary ligand ephrinA5 via targeting PI3K/Akt signaling pathways. Further understanding of the negative regulation of ligand-dependent EphA7 signaling will expand our knowledge of the molecular pathogenesis of PCa. Our discovery provides novel insight into the mode of action of Eph receptors in cancer cells and represents a new facet in the complexities of Eph receptor function.

## Materials and methods

### Plasmids and site-directed mutagenesis

Human EphA7 (GenBank accession no. NM_004440.3) cDNAs were obtained from OriGene (Rockville, MD, USA). EphA7 mutants were generated using the site-directed gene mutagenesis kit (Stratagene, La Jolla, CA, USA). Tyr597, Tyr608, Tyr614 and Tyr791 were each replaced with phenylalanine in the kinase-dead (KD) mutant. We obtained the EphA7 cytoplasmic domain truncation mutant (ΔCyto, from N-ter to Phe 576) via PCR amplification. Then, EphA7 mutants were each subcloned into the pWPXLd vector (Invitrogen, Grand Island, NY, USA). Finally, lentivirus particles were generated in HEK293T cells harboring pSPAX2/pMD2.G packaging plasmid and various forms of the pWPXLd-EphA7 plasmid.

### Cell culture, stimulation and protein overexpression

All cell lines were purchased from Chinese Academy of Sciences Cell Bank (Shanghai, China). RWPE-1 was cultured in defined keratinocyte-SFM supplemented with 5 ng/ml epidermal growth factor and 50 *μ*g/ml bovine pituitary extract (Gibco, Carlsbad, CA, USA). HEK293T, LNCaP, PC-3 and DU145 cells were grown in RPMI-1640 containing 10% FBS and 1% penicillin–streptomycin (Gibco). All cells were incubated at 37 °C in a humidified incubator containing 5% CO_2_. PC-3 and DU145 cells were stably transfected using lentivirus particles, and selected with 400 mg/l G418 for neomycin resistance. For studies of soluble ephrinA5, cells were serum deprived for 24 h and then treated with 1 *μ*g/ml Fc or ephrinA5-Fc (R&D Systems, Minneapolis, MN, USA) for the indicated times.

### Clinical samples

Fifty BPH specimens, 64r PCa tissues and 20 paired non-cancerous tissues were obtained from patients who underwent radical prostatectomy or prostate needle biopsies at the urology department of Huashan Hospital (Shanghai, China) and the Urology Department of the Affiliated Hospital of Xuzhou Medical University (Xuzhou, Jiangsu, China) between March 2013 and December 2016. All samples were verified by histological examination of sequential sections. Tumor stage was evaluated according to the 7th edition of the American Joint Committee on Cancer TNM (tumor node metastasis) classification system. Informed consent was obtained from all subjects, and the research protocol used in the present study was approved by the Ethics Committee of Huashan Hospital and the Affiliated Hospital of Xuzhou Medical University. Patient clinical and histological characteristics are listed in Supplementary Table S1.

### Quantitative real-time PCR

Total RNA was extracted and reverse transcribed with the Prim-Script RT Reagent Kit (TaKaRa, Dalian, China) according to the manufacturer’s protocol. Quantitative real-time (qRT)-PCR was performed using the ABI Prism 7500 sequence detection system (Applied Biosystems, Foster City, CA, USA) and the TaqMan Gene Expression probe: Hs00177891_ml, Hs00157342_ml and Hs99999905_ml were used to detect EphA7, ephrinA5 and GAPDH, respectively (www.appliedbiosystems.com). The PCR cycle included one cycle at 95°C for 5 min, followed by 40 cycles at 95 °C for 15 s and 60 °C for 34 s.

### IHC and imaging

The protein expression of EphA7, Ki-67, active-caspase-3 and ephrinA5 in tissue was determined using the following antibodies: rabbit anti-EphA7 polyclonal antibody (1 : 50, Abcam, Cambridge, UK), rabbit anti-Ki67monoclonal antibody (1 : 100, Cell Signaling Technology, CST, Danvers, MA, USA), rabbit anti-cleaved caspase-3 polyclonal antibody (1 : 100, CST) and goat anti-ephrinA5 polyclonal antibody (1 :  80, R&D Systems) via IHC assays as previously described.^29^

### Western blotting and immunoprecipitation

Western blotting and immunoprecipitation were performed as previously described.^30^ Briefly, cells and tissues were lysed and then immunoprecipitated with an EphA7-specific antibody (CST). The protein was separated by 10% SDS-PAGE gels and then transferred to a polyvinylidene fluoride membrane. The membrane was incubated with the following antibodies: rabbit polyclonal antibody anti-EphA7 (1 : 1000, CST), anti-EphA7(phospho Y791, 1 : 500, Abcam), anti-phospho-tyrosine (1 : 2000, CST), anti-t-Akt (1 : 2000, CST), anti-p-Akt (1 : 1000, CST) anti-pro-caspase-3 (1 : 2000, CST), anti-cleaved caspase-3 (1 : 1000, CST), anti-GAPDH (1 : 1000, CST), anti-Bcl-2 (1 : 1000, CST) and anti-Bax (1 : 1000, CST); goat polyclonal antibody anti-ephrinA5 (1 : 1000, R&D Systems); and mouse monoclonal antibody anti-*β*-actin (1 : 5000, Proteintech, Rosemont, IL, USA). After the incubation of secondary antibody, the bands were detected using ECL western detection reagents in LAS-3000 system (Fuji Film, Tokyo, Japan).

### Cell proliferation assay

PCa cells viability assay were carried out using a colorimetric WST-8 assay (Dojindo, Tokyo, Japan) as previously described.^8^

### Scratch migration assay and invasion assay

Scratch migration assay and invasion assay were carried out as previously described.^29^

### Subcutaneous xenograft tumor model

A subcutaneous xenograft tumor model was performed as previously described.^29^ Briefly, male BALB/c nude mice (4–6 weeks, 18–20 g) were purchased from the Slaccas Company (Shanghai, China) and fed under specific pathogen-free conditions. A total of 1 × 10^7^ PC-3 cells harboring EphA7(WT), EphA7(KD), EphA7(ΔCyto) or Control in 0.1 ml saline suspension were mixed with 0.1 ml of Matrigel (BD, Heidelberg, Germany) and subcutaneously injected between the scapulae of narcotized (O_2_/CO_2_) mice (six mice per group). The mice were assessed daily and weighed once a week. The tumor sizes were measured using a dial caliper in a blinded manner and tumor volumes were calculated with the following formula: volume=width^2^ × length × 0.52. All animal protocols were approved by the Shanghai Medical Experimental Animal Care Commission. When primary tumors of control mice exceeded 2 cm^3^ or ulcerated the skin (~49 days), mice were terminally narcotized and killed. Primary tumors were removed, weighed and processed for histologic examinations.

### Statistics

The differences of gene expression levels between non-cancerous prostate tissue and PCa tumor tissues, as well as BPH tissue specimens and the associations of the expression of EphA7 and ephrinA5 with clinicopathological parameters in PCa tissues were evaluated by a chi-square test. Pearson correlation coefficients (r) were calculated to evaluate the relationship between ephrinA5 expression and the ratio of pEphA7/EphA7 in PCa tissues. Other data were analyzed by Student’s *t-*test using SPSS 16.0 software (Chicago, USA). *P-*values <0.05 were considered statistically significant.

## Publisher’s Note

Springer Nature remains neutral with regard to jurisdictional claims in published maps and institutional affiliations.

## Figures and Tables

**Table 1:** Correlation of expression of EphA7 mRNA and ephrinA5 transcript with clinical and histological parameters in PCa patients

	**EphA7 mRNA expression**			**ephrinA5 mRNA expression**		
	**Normal**	**Reduced**	*P***-value**^a^	**Normal**	**Reduced**	*P***-value**^a^
*Age (years)*						
≤70	20	14	0.479	14	20	0.712
>70	15	15		11	19	
*PSA (ng/ml)*						
≤10	7	7	0.690	4	10	0.363
>10	28	22		21	29	
*Stage (TNM)*						
T1–T2	14	15	0.348	16	13	0.016
T3–T4	21	14		9	26	
*Gleason score*						
6–7	7	6	0.946	9	4	0.013
8–10	28	23		16	35	
*Prostate volume (ml)*						
≤50	25	20	0.830	18	27	0.813
>50	10	9		7	12	

Normal: 0.5≤2^-ΔΔCt^ ≤2; Reduced:2^-ΔΔCt^<0.5.

a *χ*^2^ (two-tailed).
