# Supplementary material for: Ligand-dependent EphA7 signaling inhibits prostate tumor growth and progression
Source: Cell Death Dis. 2017 Oct 12;8(10):e3122–. doi: 10.1038/cddis.2017.507 (PMC5682672; doi:10.1038/cddis.2017.507)
Supplement: Supplementary Figure 1 [file cddis2017507x1.docx]

**
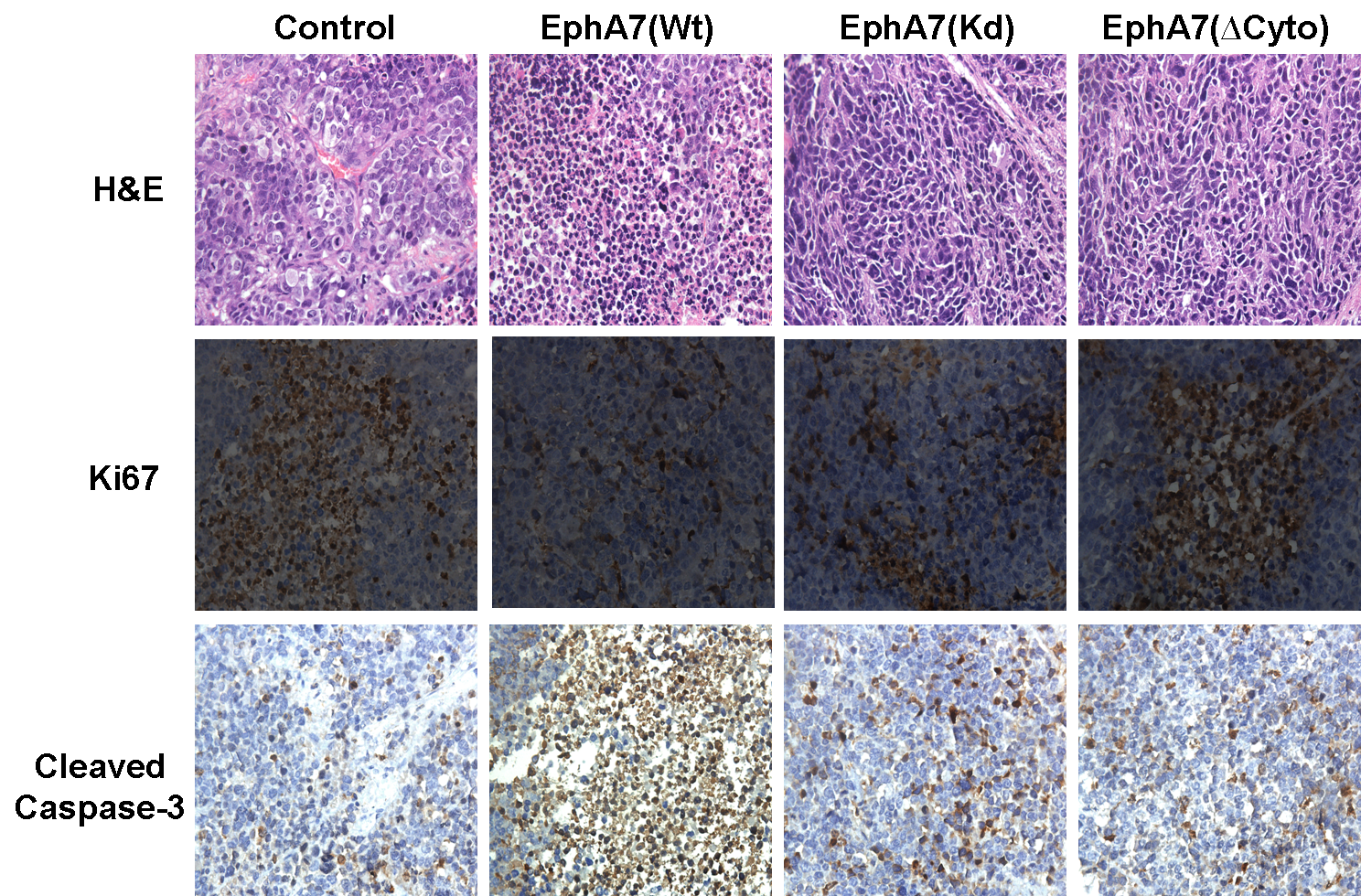
**

**Supplementary FigS1. Microscopic pathology and immunohistochemistry of graft tumor on PC-3 cells expressing wide-type** **and mutant forms of EphA7receptor.** Subcutaneous xenograft PCa models were generated using PC-3/EphA7(Wt), PC-3/EphA7(Kd), PC-3/EphA7(∆Cyto) orPC-3/Control cells. Tumors were harvested and stained with H&E, anti-Ki67 antibody and anti-cleaved caspase-3 antibody. Magnified ×400.
