# Supplementary material for: Ligand-dependent EphA7 signaling inhibits prostate tumor growth and progression
Source: Cell Death Dis. 2017 Oct 12;8(10):e3122–. doi: 10.1038/cddis.2017.507 (PMC5682672; doi:10.1038/cddis.2017.507)
Supplement: Supplementary Table 1 [file cddis2017507x2.docx]

**Table I** Correlation of expression of EphA7 mRNA and ephrinA5 transcript with clinical and histological parameters in PCa patients

|  | EphA7 mRNA expression | | *p*-Value^1^ | ephrinA5 mRNAexpression | | *p*-Value^1^ |
| --- | --- | --- | --- | --- | --- | --- |
|  | Normal Reduced | |  | Normal Reduced | |  |
| Age (years) | | | | | | |
| ≤70 | 20 | 14 | 0.479 | 14 | 20 | 0.712 |
| >70 | 15 | 15 |  | 11 | 19 |  |
| PSA (ng/ml) | | | | | | |
| ≤10 | 7 | 7 | 0.690 | 4 | 10 | 0.363 |
| >10 | 28 | 22 |  | 21 | 29 |  |
| Stage (TNM) | | | | | | |
| T1-T2 | 14 | 15 | 0.348 | 16 | 13 | 0.016 |
| T3-T4 | 21 | 14 |  | 9 | 26 |  |
| Gleason score | | | | | | |
| 6-7 | 7 | 6 | 0.946 | 9 | 4 | 0.013 |
| 8-10 | 28 | 23 |  | 16 | 35 |  |
| Prostate volume (ml) | | | | | | |
| ≤50 | 25 | 20 | 0.830 | 18 | 27 | 0.813 |
| >50 | 10 | 9 |  | 7 | 12 |  |

**Normal:0.5≤2^-△△Ct^ ≤2; Reduced：2^-△△Ct^<0.5; ^1^χ^2^(2-tailed).**
